# Supplementary material for: Lymph Node Number Predicts the Efficacy of Adjuvant Chemoradiotherapy in Node-Positive Endometrial Cancer Patients
Source: Diagnostics (Basel). 2020 Jun 4;10(6):373. doi: 10.3390/diagnostics10060373 (PMC7345621; doi:10.3390/diagnostics10060373)

Supplementary

Table S1 Univariable and multivariable Cox proportional hazards model for overall survival (*n* = 360).

Variable	Univariable		Multivariable	
	**Hazard Ratio (95% CI)**	***p*-value**	**Hazard Ratio (95% CI)**	***p*-value**
Age, continuous	1.02 (1.00–1.05)	0.05	1.00 (0.98 – 1.03)	0.98
FIGO stage				
IIIC1	Reference		Reference	
IIIC2	1.47 (0.99 – 2.19)	0.06	1.34 (0.83 – 2.16)	0.23
AJCC T-stage				
T1	Reference		Reference	
T2	1.29 (0.73–2.26)	0.38	1.12 (0.63 – 1.99)	0.71
T3	2.15 (1.36–3.39)	0.001	1.63 (1.00 – 2.65)	0.05
Histological grade and type				
Endometrioid grade 1-2	Reference		Reference	
Endometrioid grade 3	1.66 (0.96-2.86)	0.07	1.81 (1.04–3.15)	0.04
Non-endometrioid	4.17 (2.40-7.24)	< 0.001	3.57 (1.96 – 6.47)	< 0.001
Surgical type				
Modified RH	Reference		Reference	
TAH/BSO	0.64 (0.41 – 1.01)	0.06	0.67 (0.42 – 1.08)	0.10
Number of LNs removed	0.99 (0.98–1.01)	0.32	0.99 (0.97–1.00)	0.07
Number of positive LNs				
1	Reference		Reference	
2 – 5	1.44 (0.82–2.52)	0.21	1.50 (0.84 – 2.68)	0.17
≥ 6	2.36 (1.33–4.19)	0.003	1.80 (0.92 – 3.53)	0.09
Adjuvant treatment				
Chemotherapy alone	Reference		Reference	
Chemoradiotherapy	0.59 (0.39 – 0.88)	0.01	0.62 (0.41–0.93)	0.02

Abbreviations: CI, confidence interval; FIGO, International Federation of Gynaecology and Obstetrics; LN, lymph node; RH, radical hysterectomy; TAH/BSO, total abdominal hysterectomy with bilateral salpingo-oophorectom.

Supplementary Fig. S1 Landmark analysis of (A) 1-year survivors, (B) 1.5-year survivors, and (C) 2-year survivors among patients with FIGO stage IIIC receiving adjuvant chemotherapy alone or chemoradiotherapy.

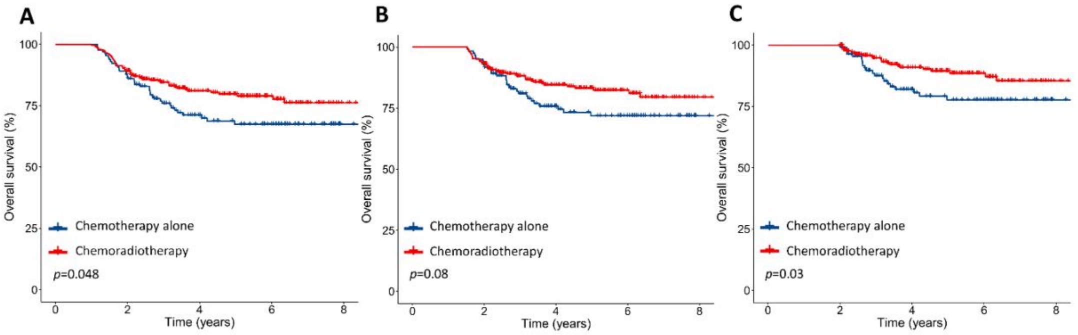

Supplementary Fig. S2 Kaplan-Meier survival curves demonstrating overall survival according to number of positive lymph nodes (A) one lymph node, (B) 2-5 lymph nodes, and (C) ≥6 lymph nodes for adjuvant chemoradiotherapy or chemotherapy alone in all patients excluding stage IIIC not otherwise specified or endometrioid carcinoma unknown grade (*n*=360).

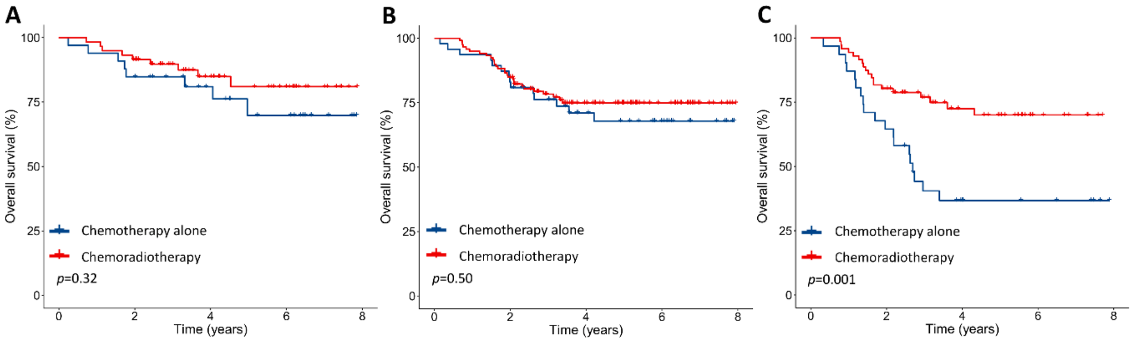

Supplement: Supplementary file 1 [file diagnostics-10-00373-s001.pdf]
